# Interacting Memory Systems—Does EEG Alpha Activity Respond to Semantic Long-Term Memory Access in a Working Memory Task?

**DOI:** 10.3390/biology4010001

**Published:** 2014-12-24

**Authors:** Barbara Berger, Serif Omer, Tamas Minarik, Annette Sterr, Paul Sauseng

**Affiliations:** 1 Department of Psychology, Ludwig-Maximilians University, Leopoldstr. 13, 80802 Munich, Germany; E-Mails: Barbara.Berger@psy.lmu.de (B.B.); Tamas.Minarik@psy.lmu.de (T.M.); 2 School of Psychology, University of Surrey, GU2 7XH Guildford, UK; E-Mails: omerserif89@gmail.com (S.O.); a.sterr@surrey.ac.uk (A.S.)

**Keywords:** beta, brain oscillations, electroencephalography, synchronisation, theta

## Abstract

Memory consists of various individual processes which form a dynamic system co-ordinated by central (executive) functions. The episodic buffer as direct interface between episodic long-term memory (LTM) and working memory (WM) is fairly well studied but such direct interaction is less clear in semantic LTM. Here, we designed a verbal delayed-match-to-sample task specifically to differentiate between pure information maintenance and mental manipulation of memory traces with and without involvement of access to semantic LTM. Task-related amplitude differences of electroencephalographic (EEG) oscillatory brain activity showed a linear increase in frontal-midline theta and linear suppression of parietal beta amplitudes relative to memory operation complexity. Amplitude suppression at upper alpha frequency, which was previously found to indicate access to semantic LTM, was only sensitive to mental manipulation in general, irrespective of LTM involvement. This suggests that suppression of upper EEG alpha activity might rather reflect unspecific distributed cortical activation during complex mental processes than accessing semantic LTM.

## 1. Introduction

Memory is a complex system which consists of various functions and cognitive processes. It can be divided into sub-systems defined by the length a mental representation is kept activated, *i.e.*, short-term memory (STM) and long-term memory (LTM). Additionally, working memory (WM) is a short-term storage system [1] which also encompasses the utilisation of information no longer available in the environment and importantly, is updated with information from LTM (episodic and semantic) in order to successfully complete the task at hand [2]. Baddeley [2,3] introduced the concept of WM being divided into two modality specific storage components (visuo-spatial sketchpad and phonological loop) which are more or less independent from LTM. In contrast, memory models by Cowan [4,5], Ruchkin and colleagues [6] or Fuster [7,8,9] assume that WM is not independent from LTM but just a subset thereof that is currently under attentional focus. A common feature of virtually all WM models, however, is the central executive (CE) which is a highly flexible attentional master component monitoring and co-ordinating all cognitive processing and is located at frontal brain areas (e.g., [3,6]).

Another consensus between the above mentioned memory models is that memory is not located at one single area in the brain but spans a vast network mainly comprising prefrontal cortex and temporal and posterior parietal areas [10,11]. Furthermore, EEG oscillatory activity has repeatedly been suggested as energy-efficient physical mechanism for temporal co-ordination of cognitive processes, locally as well as interregionally (for a review see [12]). Increase in oscillatory activity in the theta frequency range (4–8 Hz) over medial frontal brain sites (frontal midline theta, FMtheta) for instance is reported as likely candidate for representing the neural correlate of the central executive monitoring component which is essential to all memory models [13]. Sustained increase in FMtheta is suggested to reflect the active maintenance of information in WM by attentional control processes [14]. More specifically, FMtheta has been found to signal that more attentional resources and cognitive effort need to be allocated to a task (e.g., [15,16,17,18,19]).

In contrast, suppression of upper alpha (10–12/13 Hz) oscillatory activity over posterior brain areas has been reported to reflect processing of information from semantic LTM, more specifically the access to semantic LTM [20,21]; with the strength of upper alpha suppression positively correlating with the performance in tasks targeting semantic LTM specifically (for a review see [17]). Moreover, it was found that the more semantically integrated the information to be retrieved is, the more upper alpha is suppressed over posterior brain areas [22]. It was also shown that increase in upper alpha activity reflects active inhibition and protection of activated memory traces from interfering, task irrelevant LTM traces. The inhibition-timing hypothesis [23] highlights upper alpha increase and suppression as mechanism that actively inhibits interference from task-irrelevant information (upper alpha increase) and gradually releases this inhibition as access to LTM is needed to integrate information from LTM into WM in a given situation ([24,25,26,27,28] for a more recent review). Further supporting the claim of upper alpha reflecting a general gating mechanism for information from semantic LTM are findings by Sauseng and colleagues [29] who conducted a visuo-spatial delayed-match-to-sample WM experiment and showed that under increased cognitive effort upper alpha increases over posterior brain areas. Similarly, Jensen *et al.* [30] found an increase in upper alpha being correlated to load in a WM experiment. This indicates that if a situation does not require access to semantic LTM, upper alpha activity increases to prevent information from LTM being activated and from possibly interfering with the current task.

More recently oscillatory activity in the beta frequency range (13–30 Hz), which had been linked mainly to motor functions in the past, was associated with general higher cognitive processing (for a review see [31]) and memory processes [32] and specifically semantic memory [33]. Also, there is strong evidence that gamma oscillations (40–80 Hz) play an important role in information maintenance and utilisation both in humans and animal models (for a review see [34]).

However, an important but unresolved issue is how WM and semantic LTM communicate with each other since a major part of successfully completing a task is the constant update of WM with information held in LTM. The episodic buffer which forms a direct interface between episodic LTM and WM and co-ordinates the interaction between these two memory systems is fairly well studied [2]. Such direct interaction is less clear between WM and semantic LTM, however. Given the association of FMtheta with cognitive resource allocation (e.g., [16]) and monitoring of higher cognitive processes [3] FMtheta was suggested to be a prime candidate for representing the interfacing between different memory systems (for a review see [22]). More specifically, synchronisation in the theta frequency range over frontal areas indicates and monitors utilisation of information from LTM in WM at posterior brain sites ([3,17,35,36]). Kizilirmak *et al.* [37] for example showed that systematic manipulation of the complexity of the LTM retrieval processes (more specifically, LTM search) was associated with stronger slow wave negativity over the mid-frontal cortex suggesting an involvement of the anterior cingulate cortex (ACC). Since the ACC is repeatedly found as generator of FMtheta [16,35,38] this makes the assumption of FMtheta oscillations being the interface between WM and LTM all the more plausible. Similarly, Khader and Roesler [39] conducted an experiment where they systematically manipulated the number of items from different material types (objects and locations) that needed to be retrieved from LTM. They found a linear effect in both, FMtheta increase and posterior upper alpha suppression, with memory load; but only the upper alpha suppression was also sensitive to material type. They interpret their results in a way that FMtheta depicts retrieval related control processes whereas upper alpha is functionally related to the activation of information stored in LTM.

In order to investigate the oscillatory correlates of the interfacing between WM and LTM we designed a verbal WM experiment similar to the one used by Griesmayr *et al.* [40]. There, the authors used a verbal delayed-match-to-sample WM task where participants had to either retain a string of four consonant letters (maintenance) or re-arrange them according to alphabetical order (semantic manipulation with LTM access). During the delay interval they found increased FMtheta activity for the manipulation condition and attributed it to increased attentional demand. Unfortunately, Griesmayr and colleagues did not report results in the upper alpha range. In order to disentangle WM operations and semantic LTM utilisation, we added a third condition; a pure WM manipulation condition where participants had to re-arrange the consonants backwards as presented on the screen (backwards manipulation without LTM access). This should enable us to dissociate retention of information in WM from the manipulation thereof in WM. Furthermore, by comparing the two manipulation conditions we should be able to extract the neural signatures of WM accessing semantic LTM contents.

## 2. Experimental Section

### 2.1. Participants

Data were collected from 19 participants after they gave written informed consent; 11 were female (M_age_ = 20.18, SD_age_ = 1.78) and eight were male (M_age_ = 22.5, SD_age_ = 2.07). They had normal or corrected to normal vision and reported to have no prior neurological conditions. All but three were right handed (handedness was assessed with the Edinburgh Handedness Inventory, [41]). The study was approved by the University of Surrey Ethics Review Board.

### 2.2. Stimulus Presentation

Participants were comfortably seated at a standardised distance of 150 cm in front of a 19 inch Dell flat screen monitor with a total resolution of 1280 × 1024 pixels in a well lit room. Presentation Version 0.71 (Neurobehavioural Systems, Inc., Berkeley, CA, USA) was used to control visual stimulation.

### 2.3. Experimental Task and Trial Setup

**Figure 1:**
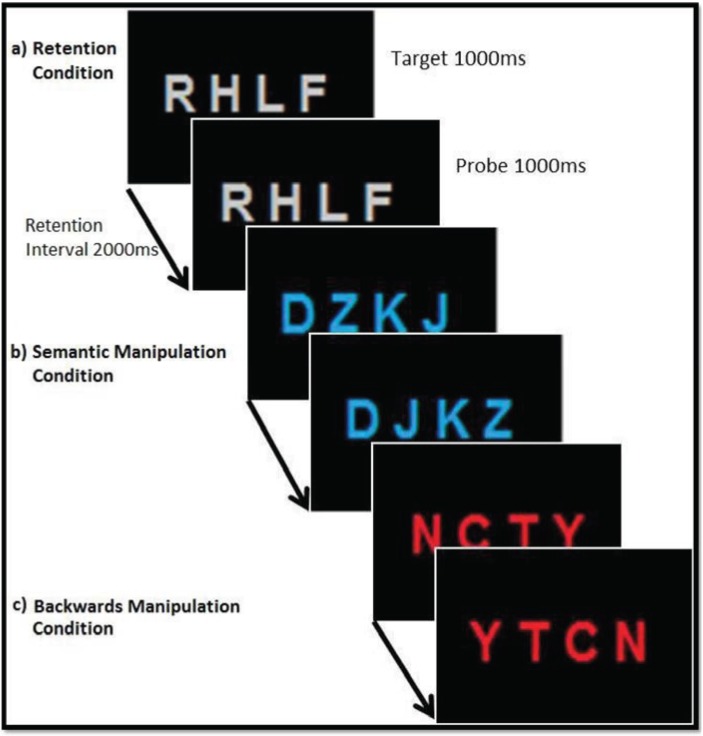
Experimental design of the delayed-match-to-sample task with examples of match trials for the conditions “retention” (RET), “semantic manipulation” (SEM) and “backwards manipulation” (BACK). Participants had to either simply retain the letter string in memory (**a**), re-arrange the letter string according to alphabetical order (**b**) or re-order the letter string backwards (**c**) during the retention interval and compare it with a probe letter string. Target presentation was 1000 ms, retention interval 2000 ms and probe presentation 1000 ms.

In the present experiment participants had to perform a verbal delayed-match-to-sample-task (see Figure 1). The verbal material consisted of visually presented consonant letter strings (target letter sting) with four items each, shown simultaneously for a period of 1000 ms against a black background centrally on the computer screen (font = Arial; font size = 80). The letters were either coloured in grey, red or blue with the colour serving as indication of which condition had to be performed in the respective trial. When the letters were grey (retention condition) participants had to simply retain the letters in their exact order during the following retention interval of 2000 ms before comparing them to a probe letter sting. When the letters were red (backwards manipulation condition), participants needed to re-order them backwards during the retention interval and retain the newly arranged letter sequence until the probe letters appeared on screen. When the letters were presented in blue (semantic manipulation condition) participants were instructed to re-arrange the letters according to alphabetical order. After the retention interval a probe letter string appeared on the monitor and participants had to decide and indicate by button press (left- or right-click on a computer mouse) whether it matched their mental representation of the target letter string (either retained or manipulated, depending on instruction) or not. The inter-trial interval varied between 2000 and 3000 ms (see Figure 1).

All participants carried out a practice run which consisted of 30 trials in total with 10 for each condition and an equal amount of match and non-match trials. The actual experiment consisted of 54 trials per condition, resulting in a total number of 162 trials with 50% of them being a match and 50% being a non-match between target and probe. All 162 trials were presented in a completely randomised order.

### 2.4. EEG Acquisition

EEG data were continuously recorded from 30 Ag-AgCl electrodes inserted into an electrode cap (Easy-cap) according to the 10-10 international system; recording sites were FP1, FP2, F7, F3, AFz, Fz, F4, F8, FC5, FC1, FC2, FC6, T3, C3, Cz, C4, T4, CP5, CP1, CP2, CP6, P7, P3, Pz, P4, P8, PO3, PO4, O1 and O2. Scalp electrodes were referenced against a ring-electrode placed on the tip of the nose and the ground electrode was placed on the forehead. EEG signals were registered with a Brain Products BrainAmp MR+ 32-channel EEG amplifier within the bandwidth of 0.016 and 80 Hz and a sampling rate of 1000 Hz. The impedance of each of the electrodes was kept below 6 kΩ. Vertical and horizontal eye movements and blinks were monitored with two electrodes placed above and next to the left eye (EOGs). Stimulus presentation and EEG acquisition were synchronised, and coded event triggers marked the onset of each stimulus, response screen and the participants’ responses in the EEG signal recorded with BrainVision Recorder software (Brain Products, Gilching, Germany).

### 2.5. EEG Analysis

For the analysis of the EEG signals Brain Vision Analyzer 2.0 (Brain Products, Gilching, Germany) was used. Data were filtered with a high- and a low-cutoff of 1 Hz and 80 Hz, respectively, and a Notch-filter was set to 50 Hz. Semi-automatic Ocular Correction with Independent Component Analysis (Ocular Correction ICA) was used to correct for eye blink artefacts. The ocular correction function as implemented in the Analyzer 2.0 is an ICA-based correction process and uses a simplified version of ICA allowing ocular artefacts in the EEG signal to be corrected specifically (for detailed information see [42]) Visual inspection of the data was then carried out in order to exclude segments which show artefacts created by muscle activity and extensive eye movements which could not be corrected with ICA.

The sampling rate of the raw EEG was changed from 1000 Hz to 1024 Hz based on spline interpolation in order to get a power of two for application of Fast Fourier Transformation later. The data recorded during the retention interval of each trial were then segmented into epochs of 1000 ms each, resulting in two time intervals per retention period (t1: from stimulus offset until 1000 ms later and t2: from 1000 ms after stimulus offset until probe onset at 2000 ms after stimulus offset). The rationale behind the subdivision of the retention interval into two separate time periods is that in the manipulation conditions the mental manipulation most likely takes place during the first half of the retention interval while retention processes like rehearsing the newly acquired letter sequence are taking place during the second half (see [40,43]). The average number of segments per condition and time period was 45.91 artefact free trials (with a minimum of 42 trials per condition). The amount of match and non-match trials that were included in the EEG analysis for each condition did not differ and hence should not influence obtained EEG results.

#### 2.5.1. Scalp Level EEG Analysis

For the analysis of the EEG signal on scalp level (as recorded by the scalp electrodes) Laplacian Current Source Density Transformation (CSD) was calculated (order of splines: 4; maximal degree of Legendre polynomials: 10; Lambda: 1 e^−5^) in order to attenuate effects of volume conduction. CSD reduces global unspecific activity while increasing local activity (for a detailed description see [44]). In order to obtain frequency power estimates (power spectra) Fast Fourier Transformation (FFT) was carried out (Hamming window of 10%) and regions of interest (ROIs) were defined (see [29,30,45,46,47,48], or for a review [49]) for the frequencies of interest; resulting in one frontal ROI (frontal-midline/FM = electrode sites AFz and Fz) and one posterior ROI (parieto-occipital/PO = electrode sites P3, Pz, P4, PO3, PO4, O1 and O2) which was later additionally divided into one right lateral posterior ROI (rPO; P4, PO4 and O2) and one left lateral posterior ROI (lPO; P3, PO3 and O1) for complementary analysis of hemispheric distribution. Frequencies of interest for this study were theta (4–7 Hz), upper alpha (10–13 Hz), lower beta (beta1, 13–20 Hz), upper beta (beta2, 20–30 Hz), slow gamma (gamma1, 30–50 Hz) and fast gamma (gamma2, 50–70 Hz). The frequency specific EEG amplitude was then collapsed over electrodes to obtain the estimates for the defined ROIs and trials were averaged for every participant within each condition for time window one and time window two separately.

#### 2.5.2. Source Level EEG Analysis

For the analysis on source level the derived scalp EEG signal was analysed using Standardized Low Resolution Electromagnetic Tomography (sLORETA; [50]) in order to estimate cortical sources of oscillatory activity in specific frequencies. The cortical grey matter and hippocampus are represented by 6430 voxels at 5 mm spatial resolution each, according to a three-shell spherical head model registered to the Talairach human brain atlas (for details see [50]). Taking volume conductance (CSD implemented into LORETA) and possible dipole structure into account and assuming that neighbouring neurons tend to fire in a synchronized fashion, sLORETA computes a current distribution throughout the full volume of the brain directly and looks for the smoothest possible 3-dimensional current distribution [51]. The raw scalp EEG is estimated back to the most likely source. The procedure for present analysis involved specification of a certain frequency band and comparison of two different conditions or time intervals with each other across participants for each single voxel. For each comparison (*t*-tests), 5000 randomisations were run in order to correct for multiple comparisons and to determine a critical *t*-value (two-tailed; see [52]). Cortical voxels exhibiting a *t*-value beyond the critical (positive or negative) *t*-value obtained in the bootsrapping procedure were defined as showing a significant difference in current source density between conditions.

### 2.6. Statistical Analysis

For statistical analysis of the scalp EEG data and behavioural data the Statistic Package of Social Sciences (IBM SPSS Version 19, IBM, Armonk, NY, USA) was used.

#### 2.6.1. Behavioural Analysis

Behavioural performance was monitored in order to assess task difficulty as higher task demand was shown to require allocation of more cognitive resources reflected by oscillatory brain activity (e.g., [30]). It is assumed that the manipulation conditions are more difficult than the simple retention condition as more cognitive effort is required for the execution of a mental manipulation than for pure retention of information. Therefore, the mean percentage of correct responses (hits and correct rejections) in each condition was calculated and statistically compared using repeated-measures ANOVA (with the factor CONDITION) after being tested for normal distribution. Paired sample *t*-tests were used for post-hoc comparisons and FDR (false discovery rate, [53]) correction was calculated to account for family-wise error rate.

#### 2.6.2. EEG Data Analysis

For statistical analysis of scalp EEG signals all trials (match and non-match, correct and incorrect) were used. Incorrect trials were included in the analysis due to the fact that their number was rather small; and secondly, incorrect responses might also be caused by erroneous encoding or retrieval processes, whereas the current analysis exclusively focused on processes during the delay interval. For statistical analysis of amplitude estimates two-way repeated-measures ANOVAs with the factors CONDITION (retention, backwards manipulation, semantic manipulation), ROI (frontal, posterior) were calculated with the Fast Fourier transformed data for each frequency band individually for the first time window of the retention interval (see EEG Analysis 2.5.). Greenhouse-Geisser corrections were applied if necessary and significance level was set to *p* < 0.05. Paired sample *t*-tests were used for post-hoc comparisons of conditions for each time interval and ROI separately for all frequency bands; FDR correction was used to account for multiple testing.

## 3. Results and Discussion

### 3.1. Behavioural Data

Performance, as measured by percentage of correct responses, was very high for all three conditions (RET (92.98%, SD 5.71), BACK (84.79%, SD 10.13), SEM (82.65%, SD 10.36)). The one-way repeated measures ANOVA comparing correct responses between the three conditions revealed a main effect of CONDITION (F(1.54, 27.71) = 14.12, *p* < 0.001, η_p_^2^ = 0.44). Post-hoc paired sample *t*-tests show that both manipulation conditions differ significantly from the retention condition with performance being higher in the retention condition (RET *vs.* BACK *t*(18) = 4.14, *p* < 0.001 and RET *vs.* SEM *t*(18) = 6.67, *p* < 0.001). Performance for the backwards manipulation and semantic manipulation conditions, on the other hand, does not differ significantly. These results indicate that the manipulation of the consonant letters was generally more difficult than the mere maintenance thereof. The nature of the manipulation on the other hand seemed to not significantly impact on task difficulty.

### 3.2. EEG Data

#### 3.2.1. Frontal Midline Theta and Distributed Theta Activity (4–7 Hz)

**Figure 2:**
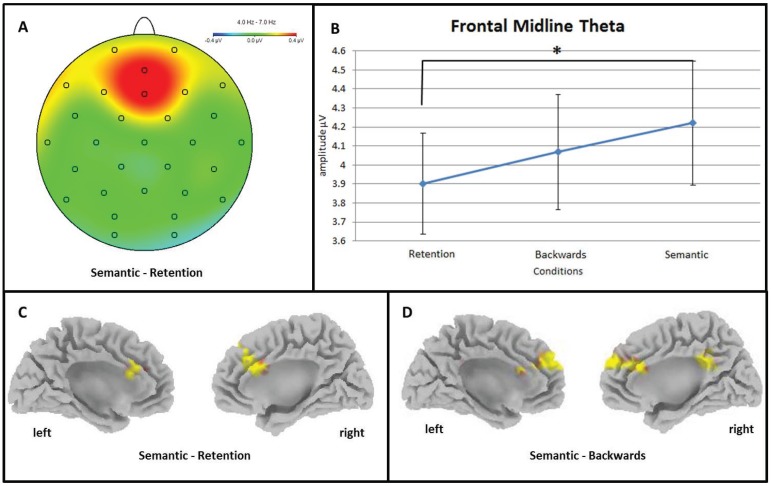
Increase in frontal midline theta (FMtheta) activity with increasing cognitive demand. (**A**) Headmap depicting significantly higher FMtheta activity over medial frontal electrode positions during semantic manipulation (SEM) of information in working memory (WM) than during the simple retention (RET) thereof; (**B**) Line chart depicting the significant amplitude difference between the retention (RET) and the semantic manipulation (SEM) conditions. Error bars show standard errors; (**C**) Standardised sLORETA cortex depicting areas with significantly higher FMtheta activity during the semantic manipulation (SEM) of information in working memory than the simple retention (RET) thereof; (**D**) Depiction of cortical areas on source level which show significantly stronger theta activity during the semantic manipulation (SEM) of information in working memory than during the backwards manipulation (BACK) thereof. Warm colours depict increase in activity and cold colours depict activity decrease.

An ANOVA comparing frontal midline theta activity (4–7 Hz) on EEG scalp level over the frontal region of interest (frontal ROI) between the retention (RET), backwards manipulation (BACK) and semantic manipulation (SEM) conditions yielded a significant main effect of CONDITION (F(2, 36) = 4.28, *p* = 0.024, η_p_^2^ = 0.19) showing an increase of frontal midline theta activity with increasing cognitive demand (Figure 2). Post-hoc paired sample *t*-tests showed that frontal midline theta activity was significantly higher in the SEM than in the RET condition (*t*(18) = −2.67, *p* = 0.016) (Figure 2A,B). Furthermore, theta activity seems to be higher in the SEM than in the RET condition at posterior areas (posterior ROI; F(2, 36) = 4.05, *p* = 0.032, η_p_^2^ = 0.18; *t*(18) = −2.67, *p* = 0.028).

On source level (sLORETA), frontal midline theta showed significant differences in anterior cingulate cortex (ACC) and medial frontal lobe (BA 9) between conditions. Current source density was higher in the SEM condition than in the RET condition (*t* > *t*_crit_ = 3.28, *p* < 0.05) (Figure 2C) and higher than in the BACK condition (*t* > *t*_crit_ = 3.32, *p* < 0.05) (Figure 2D). Additionally, the theta frequency band showed stronger activation in SEM than in BACK (*t* > *t*_crit_ = 3.32, *p* < 0.05) in the right parietal cortex (precuneus and cingulate gyrus). All *p*-values were corrected for multiple comparisons using the FDR correction approach [53].

This linear increase of FMtheta activity is well in line with existing literature highlighting the association of increase of activity in the theta frequency range over medial frontal brain sites with task difficulty and the complexity of mental operations [18]. In our task this would indicate that simple retention of four consonant letters (RET) requires less cognitive resources or involvement of medial frontal executive processing than backwards manipulation thereof (BACK); which in turn is less effort than re-arranging the consonants according to alphabetical order (SEM). In our experiment there is no specific difference in FMtheta activity between conditions that would indicate its special involvement in semantic LTM access or the interfacing between WM and semantic LTM specifically. Hence, we suggest that medial frontal theta activity is a measure of general cognitive processing effort and resource allocation depending on the complexity of the task at hand rather.

#### 3.2.2. Upper Alpha (10–13 Hz)

**Figure 3:**
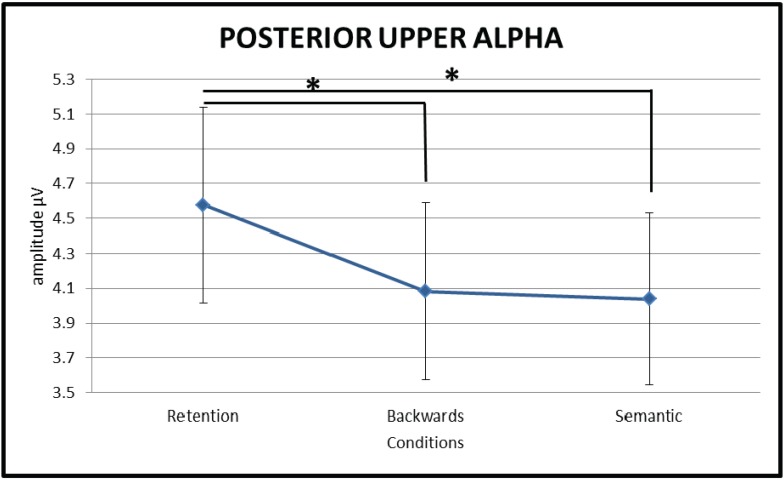
Line chart depicting the significant amplitude difference in posterior upper alpha activity between the two manipulation conditions and the retention condition. Upper alpha activity over posterior brain areas is stronger during the retention (RET) of information in working memory than during either the backwards manipulation (BACK) or the semantic manipulation (SEM) thereof. Error bars show standard errors.

A repeated measures ANOVA comparing upper alpha (10–13 Hz) oscillatory activity over posterior brain areas between the three conditions (RET, BACK and SEM) showed a significant main effect of CONDITION (F(1.224,22.03) = 8.33, *p* = 0.006, η_p_^2^ = 0.32), see Figure 3) with the retention condition (RET) showing significantly stronger upper alpha synchronisation over posterior sites than both, the backwards (BACK) (*t*(18) = 2.88, *p* = 0.01) and the semantic manipulation (SEM) (*t*(18) = 3.08, *p* = 0.006) conditions. Importantly, no significant differences were found between the backwards manipulation (BACK) and the semantic manipulation (SEM) conditions (*t*(18) = 0.479, *p* = 0.638). Moreover, source level analysis did not show any significant differences between conditions after correcting for multiple comparisons using the FDR correction approach [53].

Given the association between oscillatory activity in the upper alpha frequency range and semantic LTM access, we hypothesized that upper alpha activity over posterior brain areas would specifically respond to semantic LTM utilisation. Hence, we expected it to show a clear difference in strength of activity between the semantic manipulation condition (SEM) and the two conditions without semantic LTM access (RET and BACK). In our task no such difference was obtained but upper alpha seemed to respond to the manipulation of information in WM in general, as indicated by the significant difference between RET and BACK and RET and SEM but not between BACK and SEM. Our results suggest that suppression of oscillatory activity over posterior brain areas reflects unspecific distributed cortical activation during complex mental processes rather than access of semantic LTM specifically. This interpretation would be in line with findings by Rihs and colleagues [54] who suggest that the purpose of alpha increase during a maintenance phase over occipito-parietal areas is attention related and the inhibition of interfering information processing in general and is not necessarily related to semantic LTM. In our experiment this would indicate that the manipulation of information (BACK and SEM), irrespectively of the nature of the manipulation (with or without semantic information retrieved from LTM), needs less active inhibition of posterior cortical areas, *i.e.*, a larger posterior area/network being actively involved in the (more cognitively demanding) manipulation of information in opposition to simple retention thereof. Whereas, when the task is to simply retain four consonants (RET) a larger part of the posterior cortex can be inhibited to protect the actively maintained memory trace from interfering cortical activations.

#### 3.2.3. Lower Beta (13–20 Hz)

**Figure 4:**
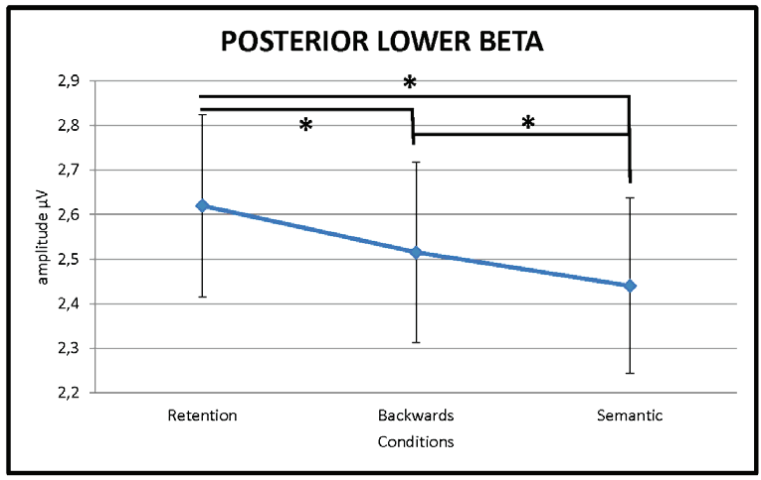
Line chart depicting the significant amplitude difference in right posterior lower beta activity between the three experimental conditions. Lower beta activity over posterior brain areas is stronger during the retention (RET) of information in working memory than during either the backwards manipulation (BACK) or the semantic manipulation (SEM) thereof. Furthermore, the backwards manipulation condition (BACK) shows significantly stronger lower beta activity than the semantic manipulation condition (SEM). Error bars show standard errors.

A repeated measures ANOVA yielded a significant main effect for CONDITION (F(1.33,24.08) = 9.86, *p* = 0.002, η_p_^2^ = 0.35)) at posterior areas of the brain in the lower beta frequency range (13–20 Hz). Post-hoc testing showed that lower beta power at posterior parts of the brain was significantly higher for the retention of the verbal material (RET) than the backwards manipulation (BACK) (*t*(18) = 2.84, *p* = 0.01) and the semantic (SEM) manipulation (*t*(18) = 3.62, *p* = 0.002) thereof. The backwards manipulation (BACK) condition and semantic manipulation (SEM) condition did not differ significantly over the posterior ROI (*t*(18) = 1.70, *p* = 0.106) but complementary analysis looking at hemispheric distribution (repeated measures ANOVA with the factor ROI split into left posterior and right posterior; (F_Condition x Region_(1.20,21.62) = 4.61, *p* = 0.037, η_p_^2^ = 0.21)) showed a significant difference between the two manipulation conditions over the right hemisphere (BACK *vs.* SEM *t*(18) = 3.94, *p* = 0.001) (Figure 4). All comparisons were FDR corrected. No significant differences between conditions were obtained on source level after FDR correction [53] was applied.

Suppression of oscillatory activity in the beta frequency range has recently been associated with higher cognitive processing outside of the motor domain [31] and has been linked to semantic LTM processing specifically [33]. Hanslmayr and colleagues [32] for example discuss the role of posterior beta frequency suppression in relation to retrieval of information from semantic LTM by reviewing studies which explicitly controlled for cognitive processes with and without semantic LTM access. They convincingly conclude that beta can not only be actively increased in order to prevent competing memories from interfering but also actively suppressed in order to promote sensory reactivation of a relevant memory. In the present study we could not find an increase or decrease in the beta frequency specifically related to semantic LTM processing but rather a linear decrease in beta activity from the retention condition (RET) via the backwards manipulation condition (BACK) to the semantic manipulation condition (SEM). We suggest that decrease in the beta frequency band at posterior brain sites more generally reflects task complexity. In accordance with this, Engel and Fries [31] argue that beta frequency oscillations seem to be related to the maintenance of a current cognitive or sensorimotor state. They reviewed literature reporting increase or decrease of oscillations in the beta frequency range and concluded that activity increase is strongly linked to the maintenance of the current status quo. Furthermore, they link abnormal enhancement in beta activity with abnormal persistence and deterioration of flexible behavioural and cognitive control. In our task this would mean that in the retention condition (RET) beta activity should be stronger than in the two manipulation conditions (BACK and SEM), which is exactly what we found. Moreover, following the hypothesis by Engel and Fries [31], the significant difference between the backwards manipulation condition (BACK) and the semantic manipulation condition (SEM) would indicate that the maintenance of the status quo is more relevant in the backwards manipulation (BACK) while the re-arrangement according to alphabetical order (SEM) requires significantly more reshuffling, *i.e.*, cognitive flexibility.

No significant differences were found on scalp or source level for the upper beta (20–30 Hz), lower gamma (30–50 Hz) or upper gamma (50–70 Hz) frequency bands after correction for multiple comparisons by applying FDR correction [53]. This could be due to the gamma activity being strongly linked to general maintenance functions in memory contexts in general [34], which did not differ significantly in our task. An alternative explanation could be that we were simply unable to pick up differences between conditions in the fast frequencies with EEG because of the spatial filtering which generally makes it harder to obtain subtle differences in the faster frequency ranges.

## 4. Conclusions

We conclude that oscillatory activity in the upper alpha frequency range might not, after all, have a clear cut role as marker for accessing semantic long-term memory and its utilisation in working memory, specifically. Instead, it reflects rather unspecific cortical activation during complex mental processes in general. Activity in the theta and the lower beta frequency range on the other hand seem to be an indicator of general cognitive effort, complexity of the mental operation at hand and cognitive flexibility. Importantly, our findings did not highlight one specific oscillatory frequency band as indicator of the interfacing between working memory and semantic long-term memory. Rather our findings suggest that the boundary between the two memory systems might be blurry, which furthermore, does not suggest a clear cut distinction between working memory and long-term memory.
